# Influence of Catalytic Infrared Radiation on the Protective Properties of Industrial Epoxy Primers

**DOI:** 10.3390/ma16196551

**Published:** 2023-10-04

**Authors:** Ivan Stojanović, Mirta Logar, Lovro Turkalj, Ivan Cindrić, Marin Kurtela, Hrvoje Franjić

**Affiliations:** 1 Chair of Materials Protection, Faculty of Mechanical Engineering and Naval Architecture, University of Zagreb, 10000 Zagreb, Croatia; ivan.stojanovic@fsb.hr (I.S.); lovro.turkalj@aquachem.hr (L.T.); i.cindric@facc.com (I.C.); marin.kurtela@fsb.hr (M.K.); 2 Končar Steel Structures Inc., 10000 Zagreb, Croatia

**Keywords:** primers, epoxy coating, infrared drying, curing degree, corrosion protection

## Abstract

The application of organic coatings is a common way of protecting metal substrates against corrosion. To dry the coating faster, catalytic infrared radiation (IR) can be applied. This paper aims to assess the differences in the physical, chemical, and corrosion properties of primer coatings dried with catalytic infrared radiation, compared to the same coatings dried under atmospheric conditions. Corrosion properties were characterized using humidity and a salt spray chamber, as well as electrochemical impedance spectroscopy (EIS), preceded by open circuit potential (OCP) measurement. Pencil hardness, cross-cut, and pull-off adhesion tests were used to compare the properties of examined primers before and after testing in the corrosion acceleration chambers. The microstructure and distribution of chemical composition were studied by scanning electron microscope (SEM) with energy-dispersive X-ray spectroscopy (EDX) together with Fourier-transform infrared spectroscopy (FTIR). Phase transitions in the coating were determined by differential scanning calorimeter (DSC). Infrared-dried primers achieved a higher curing degree. Therefore, their mechanical and corrosion properties are superior when compared to the same coatings dried under atmospheric conditions.

## 1. Introduction

As a result of the low corrosion resistance of mild steel and its broad usage in numerous industries [1], open-air structures [2], and machinery [3], it is necessary to perform preserved actions in the form of corrosion protection. Among the wide variety of approaches to corrosion protection, the use of organic coatings is one of the most practical and cost-effective ways [4] to prevent or decelerate the corrosion process [5,6].

Organic coatings are the most applied method [7], acting as a barrier layer separating the substrate material from the environment [8]. Understanding and controlling the entire painting process is crucial [9] to maximizing the durability of coatings. The process is influenced by the composition of base material [10] and proper surface preparation [11], as adhesion is crucial for the durability and performance of the protection [12], application process [13], and drying technique [14]. In the past couple of decades, the usage and subsequent emission of toxic volatile organic compounds (VOCs), which are an integral part of every solvent-based coat, has been increasingly reviewed as a problem regarding health, safety, and air pollution [15]. As a result, new regulations regarding the reduction of VOCs emerged, which oblige the organic coatings market to constantly develop new technologies [16] such as high-solid, water-borne, and IR/UV-curable coatings [1].

The epoxy primer generally consists of epoxy resin, a plasticizer, an amine/amide curing agent, and a thinner [17]. A key part of achieving good mechanical and corrosive properties is having good adhesion between the substrate and the primer layer [18]. Therefore, enhanced adhesion can be performed using suitable surface preparation. As the primer layer is the fundamental barrier between environmental conditions and substrate, it is necessary to thoroughly investigate many ways of degradation through accelerated corrosive tests [19]. With constant development and research in the coating industry, protective primer properties are being progressively improved to withstand harsh conditions for longer periods of time. Some researchers use nanomaterials to improve the properties of coatings [20,21], while others use radiation curing technologies [22,23].

A particular direction in which the development of the drying method has led is infrared (IR) energy. By applying IR technology, significantly higher drying speeds are achieved in addition to reduced gas consumption and combustion of VOCs in comparison with convection ovens [24]. IR drying does not require a contact medium to transfer heat. Hence, it dries the coating in two directions—from the outside in the form of absorption, and from the inside in the form of transmission. The IR two-way drying method significantly accelerates the chemical reactions in the coating, thus forming a final coating that is ready for use [1].

In this paper, catalytic infrared technology was used for drying two different solvent-borne epoxy primers from different manufacturers. To assess the influence of drying technology on coatings’ anti-corrosive properties, pencil, cross-cut, and pull-off adhesion tests were performed before and after the exposure to salt spray and a humidity chamber. Electrochemical impedance spectroscopy (EIS) was employed to acquire coating resistances, while the electrochemical potential state was obtained by open circuit potential (OCP). Electrochemical measurements were performed in 3.5% NaCl solution at various exposure intervals. To gain insight into more detailed microstructural examination and distribution of chemical composition, a scanning electron microscope (SEM) with energy-dispersive X-ray spectroscopy (EDX) together with Fourier-transform infrared spectroscopy (FTIR) analyses were used. A differential scanning calorimeter (DSC) was used to determine phase transitions in the coating.

## 2. Materials and Methods

Two-component (2K) epoxy-based primers from two different manufacturers, used in the power transformer industry, were tested to evaluate the influence of different drying methods on their anti-corrosive properties. Half of the samples were dried with catalytic infrared radiation and the other half under atmospheric conditions. Steel grit blasting abrasive was used for surface preparation to required cleanliness Sa 2.5, in accordance with ISO 8501-1 [25], and a medium (M) roughness degree, according to ISO 8503-1 [26]. The tested samples were mild steel plates with dimension 150 × 120 × 10 mm. Table 1 displays gloss, solids by volume, temperature resistance, time for the primer to become dry to touch (surface dry) under atmospheric conditions (20 °C), recommended thickness, and acceptable thickness for both primers used in the paper. Airless spray gun was used to apply the recommended thicknesses of the examined primers.

By virtue of their importance in climate alertness, environmentally friendly flameless catalytic infrared emitters were used to accelerate curing process of examined primers with no production of NOx and CO [29]. Gas catalytic infrared heaters convert natural gas to thermal energy in the form of IR rays [30]. The emitter had a dimension of 60 × 60 cm and a power of 6 kW. To estimate the completion of the IR drying process, a light pencil stroke was performed. The observed coating was considered sufficiently cross-linked when the pencil no longer left a trace on it.

According to ISO 2808 [31], nondestructive dry film thickness (DFT) measurement was obtained with Elcometer 456 (Elcometer Limited, Manchester, UK). Measurements were carried out on ten different positions per sample with instrument accuracy ±2.5 µm. Adhesion properties of the coating were determined using Elcometer 510 Automatic Pull-off Adhesion Gauge instrument (Elcometer Limited, Manchester, UK) with accuracy ±1% of full scale, in accordance with ISO 4624 [32]. Cross-cut test was also carried out, according to ISO 2409 [33]. Hardness of examined primers was evaluated using pencil hardness test, according to ISO 15184 [34].

Corrosion resistance in humid and salty conditions was tested by placing samples in laboratory corrosion acceleration chambers. Humidity Cabinet AB6 Model CW1302 (C&W Specialist Equipment, Belrose, NSW, Australia) and salt spray chamber Ascott S450 (Ascott Analytical Equipment Limited, Staffordshire, UK) were used. Conditions in test chambers were set according to ISO 6270-1 [35] for the humidity chamber and according to ISO 9227 [36] for the salt spray chamber. Samples were exposed for 120 h in the case of the humidity chamber and for 240 h in the case of the salt spray chamber. The samples were examined periodically to evaluate the degradation of coatings according to ISO 4628 [37]. The primers were tested for rusting (ISO 4628-3), cracking (ISO 4628-4), flaking (ISO 4628-5), and blistering (ISO 4628-2). On the samples that were in the salt spray chamber, an incision was made to assess the corrosion under the coating according to ISO 12944-6 standard [38]. According to this standard, corrosion around the scribe should not exceed more than 1.5 mm, calculated as:

(1) $$M=\frac{C-W}{2}$$

where C is the maximum width of corrosion across the scratch, and W is the original width of the scribe in millimeters.

To characterize the corrosion behavior and to determine coating protective properties, open circuit potential (OCP) and electrochemical impedance spectroscopy (EIS) analyses were conducted [39] with a VersaSTAT 3 Potentiostat/Galvanostat (AMETEK Scientific 131 Instruments, Princeton applied research, Berwyn, PA, USA). The measurements were performed in 3.5% NaCl solution, and the results were obtained after 24, 250, and 500 h of immersion at room temperature (23 ± 2) °C. This is a standard test solution, used in many papers because it simulates the marine environments since it is the average concentration of salt in the oceans [40,41,42]. The frequency range of the EIS test was 10^5^–10^−1^ Hz with 100 mV amplitude operating at ten points per decade. The electrochemical cell was composed of a coated mild steel panel acting as a working electrode, a reference saturated calomel electrode (SCE), and two graphite sticks operating as counter electrodes. The exposed surface zone of the working electrode was 19.625 cm^2^, while the surface of the counter electrodes was 25.5 cm^2^. Data results were fitted and analyzed by impedance analysis software ZSimpWin (Ametek Scientific Instruments, Oak Ridge, TN, USA). To achieve accurate results, each measurement was implemented in two replications.

The surface morphology and microstructure of differently dried primer coatings were examined by FEI Quanta FEG 250 Scanning Electron Microscope equipped with an Oxford PENTAFET detector (Oxford Instruments, Belfast, UK). The energy used for the analysis was 20 keV [43]. The surface morphology observations were fulfilled on three different cross-sections, and the representative micrograph was used.

For differential scanning calorimetry (DSC) analysis, Mettler Toledo DSC 822e (Mettler Toledo, Greifensee, Switzerland) was used. Samples of about 10 mg were analyzed in a stream of nitrogen (40 mL/min) with a heating and cooling rate of 10 °C/min in the temperature range from −100 °C to 150 °C by a double heating/cooling cycle [44,45]. The first heating cycle was used to erase the thermal history of the samples. Liquid nitrogen was used to cool the samples to low temperatures. Values of the glass transition temperature T_g_ were obtained from the second heating cycle.

**Figure 1:**
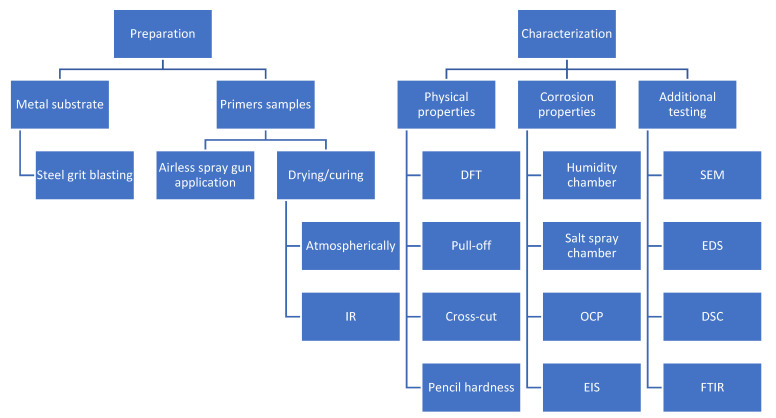
The course of experimental part of the research.

Fourier-transform infrared spectrometry (FTIR) was carried out on the sample scraped from the steel substrate to evaluate and characterize the formed primer layer. A PerkinElmer Spectrum One spectrometer (Waltham, MA, USA) with an ATR chamber (ZnSe) was used. The measurements were carried out in the wave range of 4000–650 cm^−1^ at room temperature [44,46]. The flowchart of the experimental part of the research is presented in Figure 1.

## 3. Results and Discussion

Dry film thickness (DFT), pull-off value, cross-cut, and pencil hardness results for tested primers after drying was complete, with regard to manufacturer and drying type, are presented in Table 2. All of the examined primers were applied with approximately the same thickness in order for the results to be comparable. Pull-off adhesion testing revealed higher adhesion values of Hempel’s primer, compared to Ching’s. Nevertheless, both primers exhibited very high adhesion values, since a satisfactory adhesion value is above 5 MPa, according to the ISO 19244-6 standard [38]. From the obtained results, a higher average adhesion value can be observed in the case of applying catalytic infrared radiation to dry the primer. This effect indicates a higher cross-linking degree of IR-cured primers and proves the usefulness of using catalytic infrared radiation for coating curing [44,47]. Cross-cut testing confirmed the excellent adhesion of examined primers to metallic surface, since they were rated with the best grade, zero, which is an indication that none of the squares in the grid have detached, and the edges of the cuts are completely smooth [34]. The hardness of the primers was determined using a pencil hardness test. Primer’s scratch hardness was evaluated. The hardest pencil that did not leave any mark on the sample was recorded. According to ISO 15184 [34], the hardness of the coating is classified as follows: 6H–3H, hard coatings; 2H–2B, medium hardness; and 3B–6B, soft coatings [48]. Samples from both manufacturers, dried in both ways, were rated with the same hardness rating, H, which falls into the medium hardness category.

**Figure 2:**
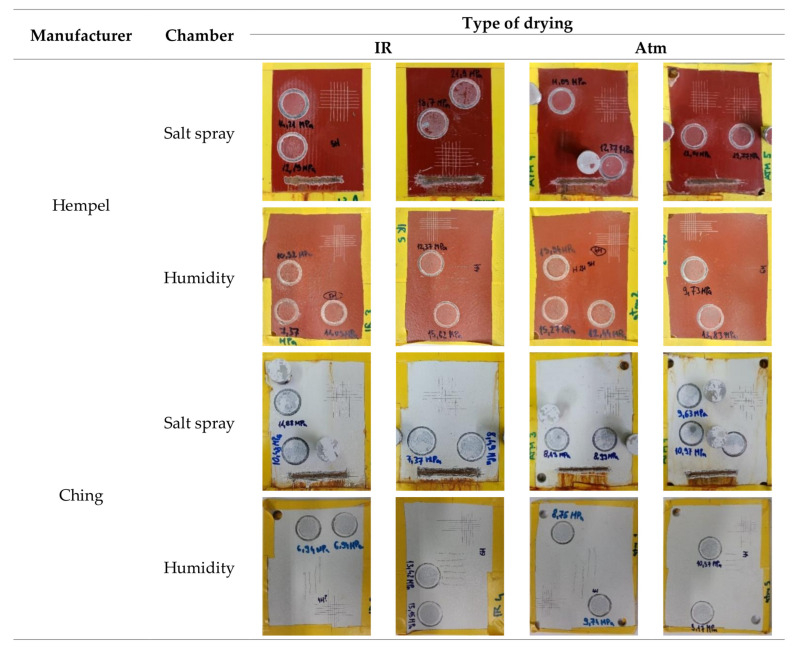
Visual appearance of the primers’ surfaces after previously defined time spent in the corrosion acceleration chambers and after their physical properties were re-examined.

After the exposure of primers to accelerated corrosion conditions in a salt spray chamber for 240 h and a humidity chamber for 120 h, the physical properties of the primers were re-examined. The visual appearance of the primers’ surfaces is shown in Figure 2. The images show two samples per manufacturer and per type of drying that were placed in the salt spray and humidity chamber. On each sample, a cross-cut test and pull-off adhesion tests were performed. Corrosion around the scribe was also performed for the samples placed in the salt spray chamber. The results of the tests are presented in Table 3 (for Hempel’s primer) and Table 4 (for Ching’s primer). They present mean DFT, cross-cut, and pull-off adhesion values as well as pencil hardness and corrosion at the scribe, M.

Pull-off values of both primers were initially higher in case of IR drying, compared to primers dried under atmospheric conditions. After the exposure to water vapor and neutral salt spray, the pull-off values of adhesion decreased but remained satisfactory. In some cases, the adhesion of IR-dried primers remained better, while, in other cases, the adhesion of atmospherically dried primers was better. The cross-cut values before placing samples in corrosion accelerating chambers were zero, which is the best grade. After the exposure, cross-cut values were rated as one which, according to the standard, is still satisfactory. The pencil hardness test showed medium hardness of the primers (H), which decreased slightly after some time spent in the chambers (primers were rated HB–H). The primers stayed in the same hardness category—medium hardness of the coating. After the exposure to humidity and neutral salt spray, no significant changes were observed in the adhesion or hardness of the primers, with regard to the drying method. For the samples placed in a salt spray chamber, the corrosion at the scribe was determined. The results show that the corrosion at the scribe is less in the case of IR-dried primers. This indicates higher cohesive strength of primers cured with the application of catalytic infrared radiation and signifies enhanced cross-linking density of IR-cured primers [49]. Nevertheless, the results were satisfactory for all tested samples. 

The samples placed in corrosion acceleration chambers were periodically examined for signs of coating degradation. Results of the primer’s rusting, cracking, flaking, and blistering after spending 240 h in the salt spray chamber and 120 h in the humidity chamber are presented in Table 5 and Table 6. After that period of time, none of the tested primers showed signs of cracking, flaking, or blistering. On the other hand, all of the samples started rusting, with a grade Ri 1, according to ISO 4628-3 [37]. No difference in degradation of the primers was observed with regard to the different method of the primer’s drying. For comparison, the tables show the times that primers spent in chambers corresponding to the times that the coating systems need to withstand in order to achieve a high degree of durability in C2—low corrosive category, medium degree of durability in C3—medium corrosive category, or low degree of durability in C4—high corrosive category, according to ISO 12944-6 [50].

The open circuit potential results, shown in Table 7, present the corrosion potential of metal substrate with applied primer coating in 3.5% NaCl solution. Corrosion potential is an indicator of the corrosion reaction from an electrochemical point of view [51]. The more negative the potential, the more likely corrosion will occur [52]. This is why more positive corrosion potential is desirable, as obtained for metallic substrate with primers dried with catalytic infrared radiation. Hempel’s IR-cured primer stabilized at −258.6 mV after 21 days in the electrolyte, while its air-dried equivalent stabilized at −522.6 mV. In the same time period, Ching’s IR-cured primer stabilized at −135.3 mV, and its air-dried equivalent stabilized at −231.9 mV. These results indicate a higher corrosion resistance of primers which are cured with catalytic infrared radiation.

Furthermore, the OCP results show that the tested primers are stable over time, since their corrosion potential, *E*_corr_, is about the same when measured at different periods of time. The exception is Ching’s primer, dried atmospherically, which decreases sharply after the first measurement, indicating a higher probability of corrosion occurring. Over time, its potential becomes more and more positive. The increase in corrosion potential was probably caused by the formation of a passive film [52]. In its data sheet, Ching states the presence of an anti-corrosive pigment, zinc phosphate. This pigment reacts with the metal substrate and forms a thin insoluble layer of zinc phosphate compounds on the surface that acts as a barrier between the metal and the surrounding environment, preventing direct contact between the metal and corrosive agents like water and ions. This process is known as passivation, and it significantly reduces the rate of corrosion [53].

**Figure 3:**
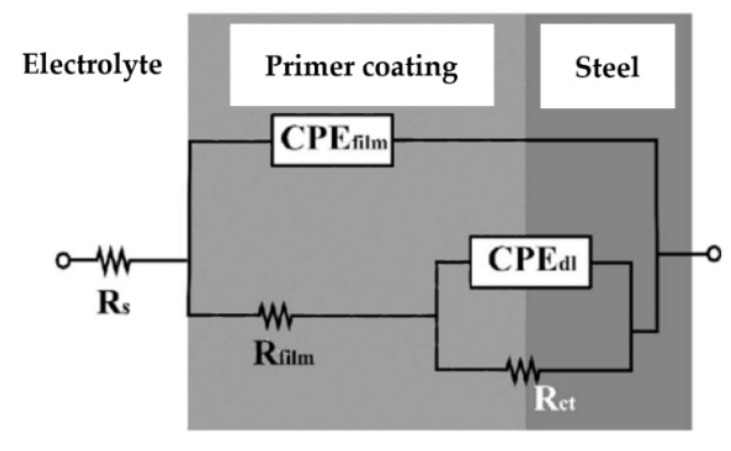
Electrical equivalent circuit model R(Q(R(QR))) [54].

To better understand the impact of infrared curing on the corrosion properties of the coatings, EIS measurements in 3.5% NaCl solution were made. The results were described with the electrical equivalent circuit model R(Q(R(QR))), depicted in Figure 3. The Figure is adapted from original work of Vallejo Vitaller, and Angst [54]. The circuit is a standard equivalent circuit used to describe organic coatings. It consists of five elements: electrolyte resistance (R_s_), coating resistance (R_film_), coating capacitance (CPE_film_), charge transfer resistance (R_ct_), and double-layer capacitance between metal and electrolyte solution (CPE_dl_). The CPE represents an imperfect, inhomogeneous system, due to surface roughness or porosity of the newly formed film, which causes an inhomogeneous charge distribution [53,54].

**Figure 4:**
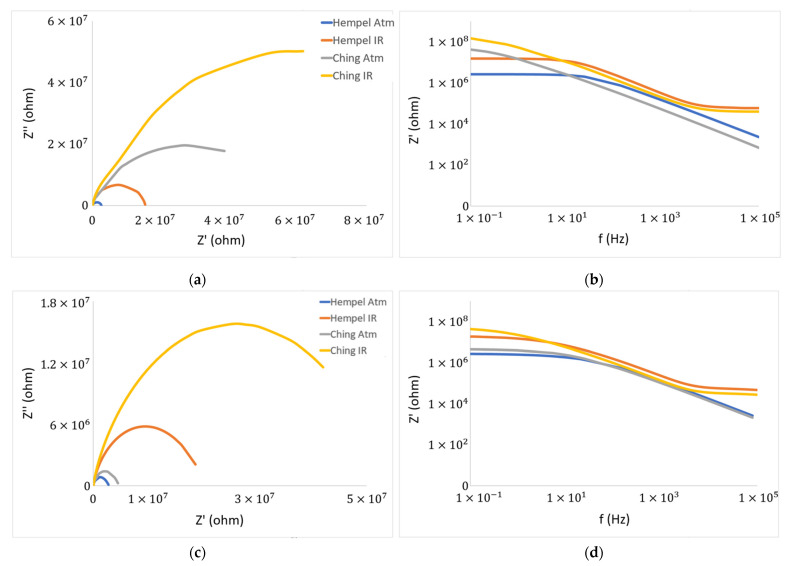
Nyquist (**a**) and Bode (**b**) plots for primers after 1 day in electrolyte; Nyquist (**c**) and Bode (**d**) plots after 21 days in the electrolyte.

For graphical illustration of EIS results, the Nyquist and Bode plots are often used. The Nyquist plot shows the relationship between imaginary impedance (Z″) and the real part of the impedance (Z′). The bigger the semicircle on the Nyquist plot, the better the resistance of the coating. On the *x*-axis of the Bode plot, the logarithmic scale of frequency is presented, while the *y*-axis impedance is shown, also in the logarithmic scale. The higher the impedance values on the Bode plot, the higher the coating’s resistance [1]. The Nyquist and Bode plots for primers after spending 1 and 21 days in the electrolyte are presented in Figure 4. The EIS spectra for the samples that spent 1 day in 3.5% NaCl solution showed the higher resistance of Ching’s primer. Both primers showed better resistance when dried with catalytic infrared radiation. In the beginning, Ching’s primers’ samples displayed an incomplete semicircle, indicating a robust coating. At this time, the electrolyte had not yet damaged the coating’s substrate, so the coating provided high resistance which the current could not penetrate [41]. Hempel’s primer displayed a full semicircle after 1 day in the electrolyte, meaning the current was able to pass through, due to lack of coating’s resistance. After spending 21 days in the electrolyte, the coatings showed a small decrease in their resistance. Ching’s IR-dried primer still has not achieved a full semicircle, indicating its great resistance. It also exhibits the largest semicircle diameter, which means it protects the metal substrate from corrosion the best [55]. Furthermore, after spending more time in the electrolyte, the IR-dried coatings show superior resistance over the atmospherically dried ones. Ching’s coating still has a slight advantage over Hempel’s, regardless of the drying method.

**Figure 5:**
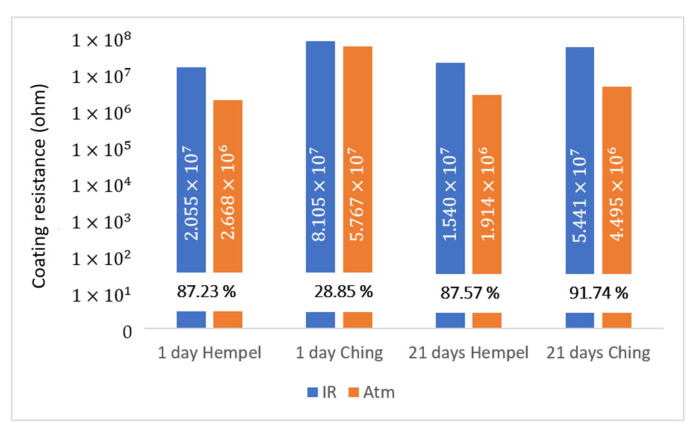
Primers’ resistance values.

Numerical values of coating resistance that were calculated according to the R(Q(R(QR))) model are displayed in Figure 5. These values were used to calculate the IR-cured coating protection efficiency, using the following equation [42]:

(2) $$IR-cured\;coating\;protection\;efficiency=\frac{\left(R_{IR-cured\;coating}-R_{Atm-cured\;coaitng}\right)}{R_{IR-cured\;coating}}\times 100\%$$

Our goal was to calculate IR-cured coating protection efficiency. As a standard specimen, we used mild steel plate covered with the same coating and dried the conventional way—under atmospheric conditions. The calculated IR-cured coating protection efficiency is shown in Figure 5 as a percentage. After the primers were immersed in the electrolyte for 1 day, the efficiency of infrared drying is significant in the case of Hempel’s primer and amounts to 87.23%. In the case of Ching’s primer, the difference between differently dried coatings is smaller, and the efficiency of infrared drying is 28.85%. After 21 days in the electrolyte, the efficiency of infrared drying compared to atmospheric drying remained approximately the same for Hempel’s coating—87.57%. In Ching’s case, the situation changed significantly. Their atmospheric-cured coating initially showed high corrosion resistance, in the same order of magnitude as infrared-cured coatings. After 21 days spent in the electrolyte, its resistance decreased by an order of magnitude and thus came on par with other atmospherically dried coatings. Ching’s IR-cured coating retained its high resistance, and the infrared drying efficiency was calculated as 91.74%. In general, the primers of both manufacturers retained their initial protective properties, especially in the case of infrared drying. Infrared drying proved to be constant, as the resistance of both manufacturers’ IR-dried coatings decreased very little over time, while atmospheric drying of Ching’s coating showed differences in resistance with respect to the time spent in the electrolyte.

Given that two industrial primers were tested, the exact composition of the coatings is not known. Better understanding of primer’s composition was sought using scanning electron microscopy together with dispersive X-ray microanalysis. SEM provided detailed images of the sample’s cross-section, enabling the study of the material’s structure and behavior on a microscopic scale.

**Figure 6:**
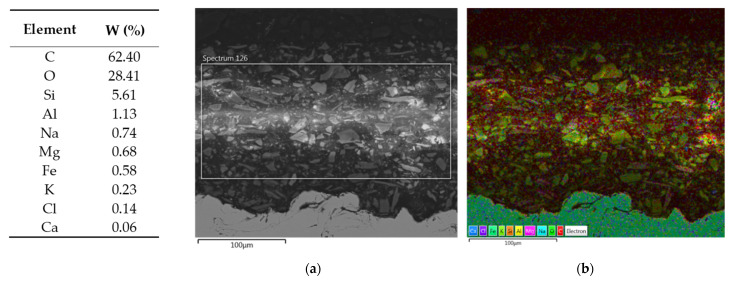
Results of SEM/EDS analysis—summary map of Hempel’s primer cross-section. The elements found in EDS field shown in (**a**) and their mass fractions are extracted in a table on the left. In (**b**), different elements are shown in different colors on the cross-section of Hempel’s primer.

**Figure 7:**
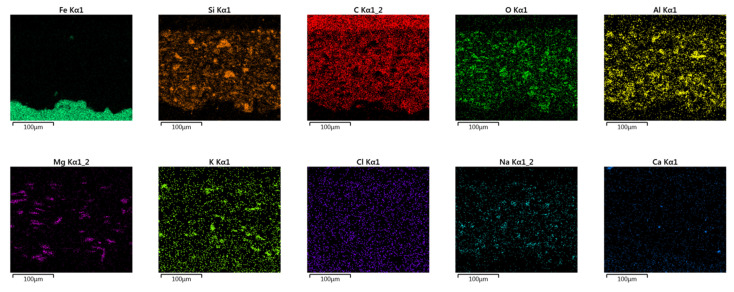
Elemental maps of Hempel‘s primer cross-section.

**Figure 8:**
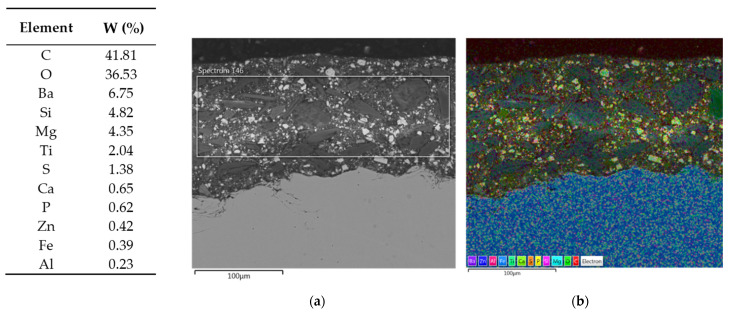
Results of SEM/EDS analysis—summary map of Ching’s primer cross-section. The elements found in EDS field shown in figure (**a**) and their mass fractions are extracted in a table on the left. In figure (**b**), different elements are shown in different colors on the cross-section of Ching’s primer.

**Figure 9:**
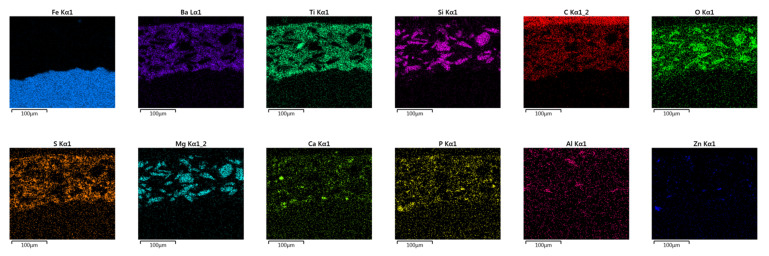
Elemental maps of Ching‘s primer cross-section.

EDS assisted in determining the elemental composition of a sample, which is essential for characterizing unknown materials and assessing the composition of complex samples. More specifically, the SEM/EDS combination helps to define chemical composition of a primer, providing insights into particle size and distribution [56,57]. Figure 6 presents the results of SEM/EDS cross-section summary map for Hempel’s primer with the elemental maps shown in Figure 7. Figure 8 and Figure 9 present Ching’s equivalents.

Figure 6 and Figure 8 also contain a table with mass fractions of elements found in the selected EDS fields. According to EDS analysis, the largest mass fraction of both primers is occupied by carbon, followed by oxygen, which is not unusual, since we are looking at organic, epoxy coatings. Furthermore, Hempel’s primer has a high proportion of silicon and aluminum, which could indicate the presence of a frequently used filler—kaolin, Al_2_Si_2_O_5_(OH)_4_. EDS of Ching’s primer showed a high mass fraction of barium, which could indicate BaSO_4_ filler, since the presence of sulfur is also proven by EDS analysis. An elemental map of Ching’s primer for titanium shows that it was also used as a filler. While it is primarily known as a pigment, titanium dioxide is also used as a filler in some paint formulations. TiO_2_ can provide barrier protection and enhance UV resistance. The presence of calcium in both manufacturers’ primers could indicate the use of CaCO_3_ filler [48]. Regarding pigments, the presence of magnesium oxide, MgO, can be read from Hempel’s, as well as Ching’s EDS. MgO is an alkaline oxide that can neutralize acidic substances, effectively slowing down corrosion reactions. Ching’s EDS reveals the possible presence of MgO/CaSiO_3_ core-shell pigments, also known as MgO shell-centering silicate cores, probably wollastonite, CaSiO_3_ [58]. It has already been mentioned that Ching’s primer contains zinc phosphate, Zn_3_(PO_4_)_2_, as a pigment, the existence of which has now been proven by EDS. This pigment, with its inhibiting effects, contributes to the high corrosion resistance of the coating [50]. The SEM/EDS combination also provides insight into the size of the particles as well as their distribution. The particles in both primers look well distributed, with no signs of agglomeration.

**Figure A1:**
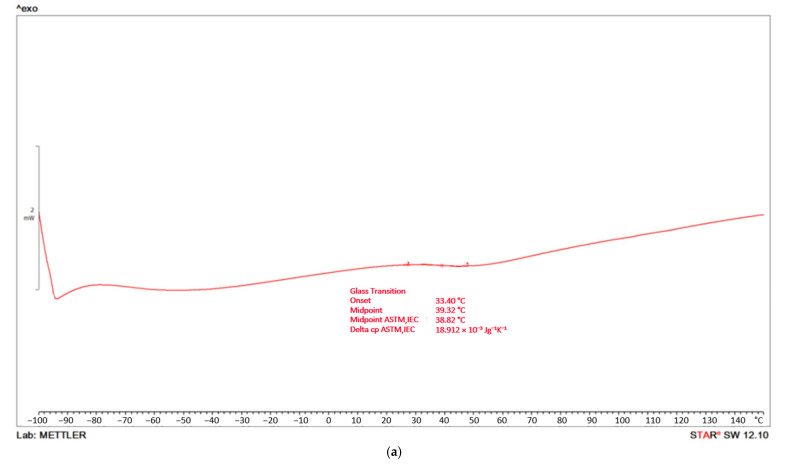

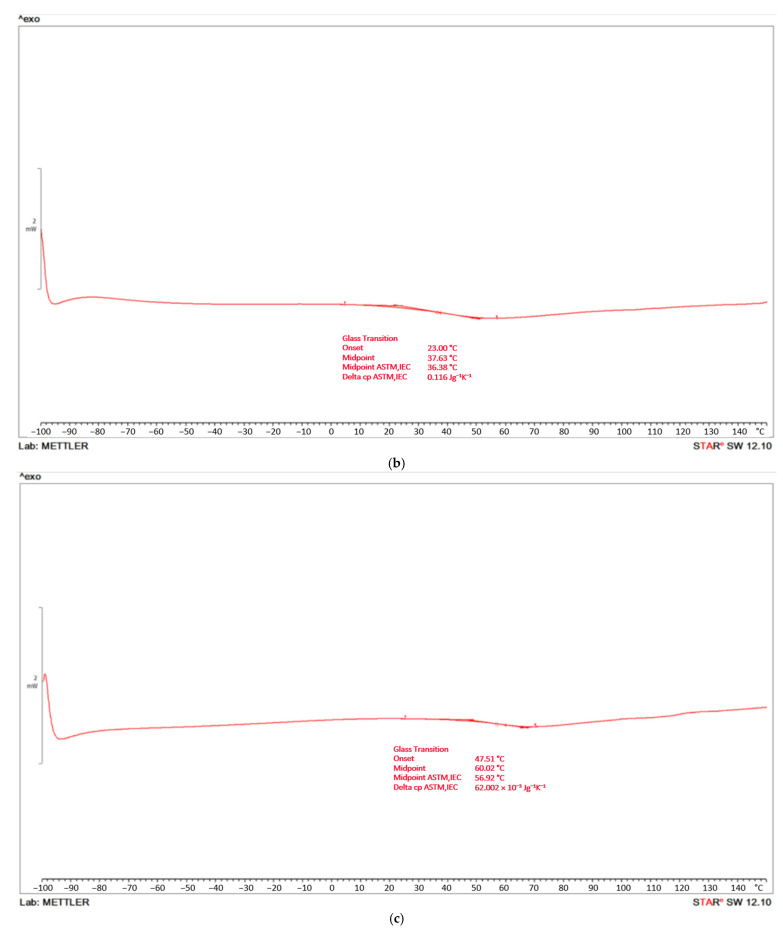

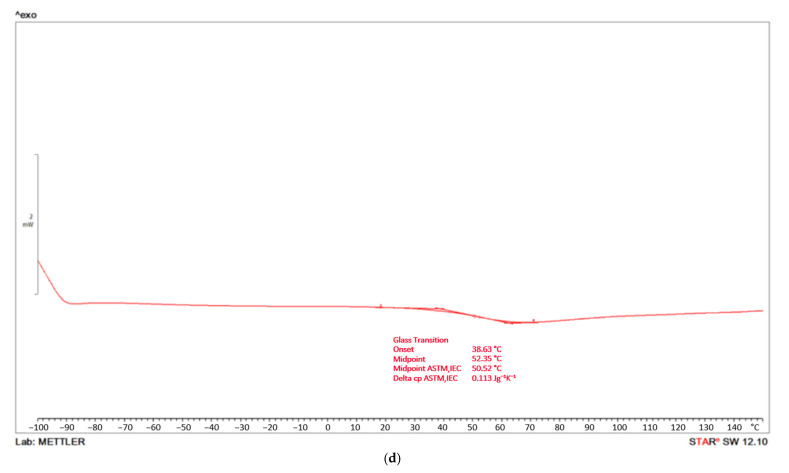
DSC thermograms of Hempel’s primer cured with catalytic infrared radiation (**a**) and under atmospheric conditions (**b**); DSC thermograms of Ching’s primer cured with catalytic infrared radiation (**c**) and under atmospheric conditions (**d**).

Better mechanical and corrosion properties of infrared-cured coatings were attributed to a higher degree of cross-linking of the coatings cured this way. This assumption was verified using DSC and FTIR analysis. Values of the glass transition temperature, T_g_, obtained from the second heating cycle during a DSC analysis, are presented in Table 8. As the primer cures, the polymer’s network mobility decreases, leading to an increase in crystallinity. The higher the crystallinity, the higher the glass transition temperature. Therefore, higher T_g_ indicates a higher curing degree of a polymer [59,60]. For Hempel’s primer, the glass transition temperature changes from 36.4 °C (for atm-cured primer) to 38.8 °C for IR-cured primer. Furthermore, Ching’s primer exhibits a significant difference in glass transition temperatures. Its value when dried atmospherically is 50.5 °C, which increases to 56.9 °C when catalytic infrared radiation is applied to cure the primer. The benefit of using catalytic infrared radiation for primer curing is evident. DSC thermograms for the second heating cycle are shown in Figure A1.

**Figure 10:**
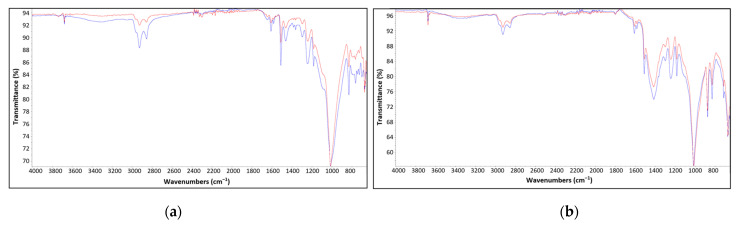
FTIR spectra of (**a**) Hempel‘s primer and (**b**) Ching‘s primer. Spectra of IR-dried primers is colored red, while blue FTIR spectra represents atmospherically dried primers.

FTIR spectra for Hempel‘s and Ching‘s primer dried atmospherically and with the application of catalytic infrared radiation are presented in Figure 10. The spectra obtained for IR-dried primers are colored red, while the spectra obtained for atmospherically dried primers are colored blue. Table 9 provides an overview of the peak positions recorded by ATR-FTIR and a description of the characteristic absorption bands for epoxy-based coatings [61,62,63,64]. No significant differences were observed between the same coating dried in different ways, since FTIR peaks were recorded at almost identical wavelengths.

## 4. Conclusions

This paper investigates the influence of applying catalytic infrared radiation to cure industrial primers from two different manufacturers. Overall better mechanical and corrosion properties of infrared-dried primers indicate that they achieved a higher curing degree. Our conclusions based on individual experiments are as follows:

- Pull-off and cross-cut tests revealed great adhesion of both primers. The adhesion was even better when catalytic infrared radiation was applied.
- Catalytic infrared radiation did not affect the primers’ hardness or their results from the corrosion acceleration chambers, in terms of cracking, flaking, and blistering.
- Corrosion at the scribe from the experiments in a salt spray chamber revealed higher cohesive strength of primers cured with catalytic infrared radiation.
- OCP and EIS results indicate a higher corrosion resistance of IR-cured primers. IR-cured coating protection efficiencies are significant and amount to around 90%.
- SEM/EDS helped to better understand specific properties of individual primers.
- DSC analysis confirmed a higher curing degree of IR-cured primers, while the results of FTIR analysis revealed no significant differences between differently dried primers.

## Figures and Tables

**Table 1:** Primer types [27,28].

Manufacturer	Hempel	Ching
Gloss	Semi-gloss	Matte
Solids by volume (Vol. %)	85 ± 2	59
Temperature resistance (°C)	120	130
Surface dry time (h)	3	3
Recommended thickness (µm)	150	60–100
Acceptable thickness (µm)	100–225	60–200

**Table 2:** Dry film thickness (DFT), pull-off value, cross-cut, and pencil hardness results for tested primers after drying was complete.

Manufacturer	Type of Drying	DFT (μm)	Pull-Off (MPa)	Cross-Cut	Pencil Hardness
Hempel	IR	165	21.25	0	H
	Atm	153	16.64	0	H
Ching	IR	164	14.88	0	H
	Atm	155	13.47	0	H

**Table 3:** Physical properties of Hempel’s primer after 240 h in salt spray chamber and 120 h in humidity chamber.

Chamber	Type of Drying	DFT_mean_ (µm)	Pull-Off (MPa) ISO 4624	Cross-Cut ISO 2409	Pencil Hardness ISO 15184	M (mm) ISO 12944-6
Salt spray	IR	168 (±2.5)	13.2 (±0.4)	1	HB	0.01
		162 (±2.5)	20.3 (±0.4)	1	F	0.11
	Atm	173 (±2.5)	12.39 (±0.4)	1	F	0.2
		170 (±2.5)	11.73 (±0.4)	1	H	0.23
Humidity	IR	135 (±2.5)	9.57 (±0.4)	1	HB	/
		141 (±2.5)	14 (±0.4)	1	HB	/
	Atm	141 (±2.5)	13.88 (±0.4)	1	HB	/
		149 (±2.5)	11.78 (±0.4)	1	F	/

**Table 4:** Physical properties of Ching’s primer after 240 h in salt spray chamber and 120 h in humidity chamber.

Chamber	Type of Drying	DFT_mean_ (µm)	Pull-Off (MPa) ISO 4624	Cross-Cut ISO 2409	Pencil Hardness ISO 15184	M (mm) ISO 12944-6
Salt spray	IR	133 (±2.5)	11.18 (±0.4)	1	H	0.02
		151 (±2.5)	7.93 (±0.4)	1	F	0.04
	Atm	142 (±2.5)	8.71 (±0.4)	1	F	0.01
		131 (±2.5)	10.3 (±0.4)	1	H	0.26
Humidity	IR	123 (±2.5)	6.84 (±0.4)	1	HB	/
		102 (±2.5)	13.29 (±0.4)	1	F	/
	Atm	128 (±2.5)	9.25 (±0.4)	1	HB	/
		129 (±2.5)	9.87 (±0.4)	1	HB	/

**Table 5:** Rusting, cracking, flaking, and blistering of Hempel’s primer after 240 h in salt spray chamber and 120 h in humidity chamber.

Chamber	Type of Drying	Rusting ISO 4628-3	Cracking ISO 4628-4	Flaking ISO 4628-5	Blistering ISO 4628-2
Salt spray	IR	Ri 1	0(S0)	0(S0)	0(S0)
		Ri 1	0(S0)	0(S0)	0(S0)
	Atm	Ri 1	0(S0)	0(S0)	0(S0)
		Ri 1	0(S0)	0(S0)	0(S0)
Humidity	IR	Ri 1	0(S0)	0(S0)	0(S0)
		Ri 1	0(S0)	0(S0)	0(S0)
	Atm	Ri 1	0(S0)	0(S0)	0(S0)
		Ri 1	0(S0)	0(S0)	0(S0)

**Table 6:** Rusting, cracking, flaking, and blistering of Ching’s primer after 240 h in salt spray chamber and 120 h in humidity chamber.

Chamber	Type of Drying	Rusting ISO 4628-3	Cracking ISO 4628-4	Flaking ISO 4628-5	Blistering ISO 4628-2
Salt spray	IR	Ri 1	0(S0)	0(S0)	0(S0)
		Ri 1	0(S0)	0(S0)	0(S0)
	Atm	Ri 1	0(S0)	0(S0)	0(S0)
		Ri 1	0(S0)	0(S0)	0(S0)
Humidity	IR	Ri 1	0(S0)	0(S0)	0(S0)
		Ri 1	0(S0)	0(S0)	0(S0)
	Atm	Ri 1	0(S0)	0(S0)	0(S0)
		Ri 1	0(S0)	0(S0)	0(S0)

**Table 7:** Open circuit potential results of primers after spending 1, 4, 7, 10, 15, and 21 days in 3.5% NaCl solution.

Day	*E*_corr_ vs. SCE (mV)			
	Hempel		Ching	
	IR	Atm	IR	Atm
1	−135.9	−419.9	−163.2	−261.9
4	−226.3	−506.1	−147.2	−476.0
7	−224.8	−488.3	−148.1	−380.6
10	−221.4	−523.9	−145.4	−362.5
15	−209.8	−516.8	−136.8	−331.8
21	−258.6	−522.6	−135.3	−231.9

**Table 8:** DSC analysis results.

Manufacturer	Type of Drying	T_g_ (°C)
Hempel	IR	38.8
	Atm	36.4
Ching	IR	56.9
	Atm	50.5

**Table 9:** Peak positions recorded on the ATR-FTIR spectra and characteristic absorption bands for epoxy-paint explained [61,62,63,64].

Wavenumber (cm^−1^)				Assignment
Hempel		Ching		
IR	Atm	IR	Atm	
3676.89	3676.86	3676.58	3676.67	O-H stretching
	3305.69	3368.37	3346.83	N-H stretching
2924.63	2925.18	2925.21	2925.19	C-H stretching of CH_2_
2853.78	2852.87	2854.77	2855.27	CH_2_ symmetrical and asymmetrical stretching
1607.22	1607.47	1606.62	1606.85	C=C aromatic cycle stretching, N-H bending of primary amine
1581.12	1581.61	1581.39	1581.91	C=C aromatic cycle stretching, N-H bending of primary amine
1508.71	1508.80	1507.71	1508.18	C-C stretching of aromatic cycle
1456.10	1460.63	1413.19	1412.01	C–H bending of CH_2_
1294.63	1294.79	1297.39	1296.65	C-O symmetric stretching of phenolic ether
1241.27	1243.25	1243.16	1241.35	C-O symmetric stretching of phenolic ether
1180.68	1181.17	1181.05	1181.13	C-O symmetric stretching of phenolic ether
1011.91	1010.74	1011.29	1012.78	C-O symmetric stretching of aliphatic ether
		873.07	872.42	C=C bending
827.86	827.62	827.48	827.46	C–H out-of-plane bending of aromatic cycle
762.67	761.10			C–H out-of-plane bending of aromatic cycle
724.75	723.25	711.46	711.25	C–H out-of-plane bending of aromatic cycle
671.37	670.49	669.75	670.42	C–H out-of-plane bending of aromatic cycle

## Data Availability

Not applicable.

## References

[1] Stojanović I., Cindrić I., Janković L., Šimunović V., Franjić H. (2022). Performance Assessment of Differently Dried Coating Systems for Potential Application in the Power Transformer Industry. Coatings.

[2] De la Fuente D., Díaz I., Simancas J., Chico B., Morcillo M. (2011). Long-term atmospheric corrosion of mild steel. Corros. Sci..

[3] Popoola A.P.I., Loto C.A., Osifuye C.O., Aigbodion V.S., Popoola O.M. (2016). Corrosion and wear properties of Ni-Sn-P ternary deposits on mild steel via electroless method. Alex. Eng. J..

[4] Bhuvaneswari T.K., Jeyaprabha C., Arulmathi P. (2022). Corrosion inhibition of mild steel in hydrochloric acid by leaves extract of *Tephrosia purpurea*. J. Adhes. Sci. Technol..

[5] Židov B., Lin Z., Stojanović I., Xu L. (2021). Impact of inhibitor loaded mesoporous silica nanoparticles on waterborne coating performance in various corrosive environments. J. Appl. Polym. Sci..

[6] Song G.-L., Feng Z. (2020). Modification, Degradation and Evaluation of a Few Organic Coatings for Some Marine Applications. Corros. Mater. Degrad..

[7] Chiba M., Tsuji Y., Takada R., Eguchi Y., Takahashi H. (2023). Formation of Self-Healing Organic Coatings for Corrosion Protection of Al Alloys by Dispersion of Spherical and Fibrous Capsules. Materials.

[8] Hu R.-G., Zhang S., Bu J.-F., Lin C.-J., Song G.-L. (2012). Recent progress in corrosion protection of magnesium alloys by organic coatings. Prog. Org. Coat..

[9] Sugimoto T., Yamaguchi K., Higashiyama Y. Detection of Paint Curing by Non-contact Surface Resistivity Measurement. Proceedings of the Joint Electrostatics Conference.

[10] Fink-Jensen P. (1965). Hardness testing of organic coatings. J. Pure Appl. Chem..

[11] Stojanović I., Alar V., Mikšić B.A., Boršić I.R. The effect of VpCI chemical pre-treatment on adhesion of organic coatings. Proceedings of the Eurocorr.

[12] Lyon S.B., Bingham R., Mills D.J. (2017). Advances in corrosion protection by organic coatings: What we know and what we would like to know. Prog. Org. Coat..

[13] Bierwagen G.P. (1996). Reflections on corrosion control by organic coatings. Prog. Org. Coat..

[14] Ouarga A., Noukrati H., Iraola-Arregui I., Elaissari A., Barroug A., Youcef H.B. (2020). Development of anti-corrosion coating based on phosphorylated ethyl cellulose microcapsules. Prog. Org. Coat..

[15] De Meijer M. (2001). Review on the durability of exterior wood coatings with reduced VOC-content. Prog. Org. Coat..

[16] Faccini M., Bautista L., Soldi L., Escobar A.M., Altavilla M., Calvet M., Domènech A., Domínguez E. (2021). Environmentally Friendly Anticorrosive Polymeric Coatings. Appl. Sci..

[17] Zin I.M., Howard R.L., Badger S.J., Scantlebury J.D., Lyon S.B. (1998). The mode of action of chromate inhibitor in epoxy primer on galvanized steel. Prog. Org. Coat..

[18] Mohseni M., Mirabedini M., Hasemi M., Thompson G.E. (2006). Adhesion performance of an epoxy clear coat on aluminum alloy in the presence of vinyl and amino-silane primers. Prog. Org. Coat..

[19] Poelman M., Olivier M.-G., Gayarre N., Petitjean J.-P. (2005). Electrochemical study of different ageing tests for the evaluation of a cataphoretic epoxy primer on aluminium. Prog. Org. Coat..

[20] Bagale U.D., Desale R., Sonawane S.H., Kulkarni R.D. (2018). An Active Corrosion Inhibition Coating of Two Pack Epoxy Polyamide System using Halloysite Nanocontainer. Prot. Met. Phys. Chem. Surf..

[21] Khodaei P., Shabani-Nooshabadi M., Behpour M. (2019). Epoxy-Based nanocomposite coating reinforced by a zeolite complex: Its anticorrosion properties on mild steel in 3.5 wt% NaCl media. Prog. Org. Coat..

[22] Roose P., Fallais I., Vandermiers C., Olivier M.-G., Poelman M. (2009). Radiation curing technology: An attractive technology for metal coating. Prog. Org. Coat..

[23] Yuan Y., Pan S., Wang T., Xia L., Liu Y., Wang X., Li L., Wnag T. (2023). Experimental and Numerical Investigations on Curing a Polyester-Based Powder Coating by Catalytic Infrared Radiation. Appl. Sci..

[24] Schmitz C., Strehmel B. (2019). NIR LEDs and NIR lasers as feasible alternatives to replace oven processes for treatment of thermal-responsive coatings. J. Coat. Technol. Res..

[25] (2007). Preparation of Steel Substrates before Application of Paints and Related Products—Visual Assessment of Surface Cleanliness—Part 1: Rust Grades and Preparation Grades of Uncoated Steel Substrates and of Steel Substrates after Overall Removal of Previous Coatings.

[26] (2012). Preparation of Steel Substrates before Application of Paints and Related Products—Surface Roughness Characteristics of Blast-Cleaned Steel Substrates—Part 1: Specifications and Definitions for ISO Surface Profile Comparators for the Assessment of Abrasive Blast-Cleaned Surfaces.

[27] Hempel “Hempaprime Multi 500”, Product Data Sheet.

[28] Ching “ESD 182 K-DB”, Product Data Sheet.

[29] Khamis M., Subramanyam B., Dogan H., Gwirtz J.A. (2011). Flameless catalytic infrared radiation used for grain disinfestation does not affect hard red winter wheat quality. J. Stored Prod. Res..

[30] Al-Dabbas M.A. (2011). Heating by Catalytic Gas Infrared Rays. Energy Eng..

[31] (2019). Paints and Varnishes—Determination of Film Thickness.

[32] (2016). Paints and Varnishes—Pull-off Test for Adhesion.

[33] (2016). Paints and Varnishes—Cross-Cut Test.

[34] (2020). Paints and Varnishes—Determination of Film Hardness by Pencil Test.

[35] (2017). Paints and Varnishes—Determination of Resistance to Humidity—Part 1: Condensation (Single-Sided Exposure).

[36] (2017). Corrosion Tests in Artificial Atmospheres—Salt Spray Tests.

[37] (2016). Paints and Varnishes—Evaluation of Degradation of Coatings—Designation of Quantity and Size of Defects, and Intensity of Uniform Changes in Appearance.

[38] (2018). Paints and Varnishes—Corrosion Protection of Steel Structures by Protective Paint Systems—Part 6: Laboratory Performance Test Methods.

[39] Jianguo L., Gaoping G., Chuanwei Y. (2005). EIS study of corrosion behaviour of organic coating/Dacromet composite systems. Electrochim. Acta.

[40] Wright J.M., Colling A. (1995). Seawater: Its Composition, Properties and Behaviour.

[41] Aleksandrov Fabijanić T., Šnajdar M., Kurtela M., Šimunović V., Marciuš M., Klaić M. (2023). Corrosion Resistance of Nanostructured Cemented Carbides with Alternative FeNi and FeNiCo Binders. Nanomaterials.

[42] Samardžija M., Alar V., Špada V., Stojanović I. (2022). Corrosion Behaviour of an Epoxy Resin Reinforced with Aluminium Nanoparticles. Coatings.

[43] Stojanović I., Cindrić I., Turkalj L., Kurtela M., Rakela-Ristevski D. (2022). Durability and Corrosion Properties of Waterborne Coating Systems on Mild Steel Dried under Atmospheric Conditions and by Infrared Radiation. Materials.

[44] Stojanović I., Logar M., Fatović I., Alar V., Rakela-Ristevski D. (2023). Experimental Study of Atmospherically and Infrared-Dried Industrial Topcoats. Coatings.

[45] Li S., Liu Z., Hou L., Chen Y., Xu T. (2020). Effect of polyether/polyester polyol ratio on properties of waterborne two component polyurethane coatings. Prog. Org. Coat..

[46] Šoić I., Martinez S., Dubravić M. (2019). Gel-Electrolyte EIS setup used for probing of IR Dried/Cured industrial coatings. Prog. Org. Coat..

[47] Uday M., Kiran Kumar P. (2020). Heat transfer studies for infrared radiation assisted curing in polymer composites. J. Phys. Conf. Ser..

[48] Kralj M., Pavković K., Stojanović I., Anđal J. (2019). Adhesion and anticorrosive properties of DTM coating as related to primer coating. Građevinar.

[49] Wei H., Xia J., Zhou W., Zhou L., Hussain G., Li Q., Ostrikov K. (2022). Adhesion and cohesion of epoxy-based industrial composite coatings. Compos. Part B Eng..

[50] Selemix A Guide to the Updated ISO 12944 Norm. https://en.selemix.com/en/technical-support/expert-know-how/a-guide-to-the-updated-iso-12944-norm/#.

[51] Kano K., Hagiwara S., Igarashi T., Otani M. (2021). Study on the free corrosion potential at an interface between an Al electrode and an acidic aqueous NaCl solution through density functional theory combined with the reference interaction site model. Electrochim. Acta.

[52] Hiromoto S., Niinomi M. (2010). Corrosion of metallic biomaterials. Metals for Biomedical Devices.

[53] Kalendova A., Veselý D., Kalenda P. (2006). A study of the effects of pigments and fillers on the properties of anticorrosive paints. Pigment. Resin Technol..

[54] Vallejo Vitaller A., Angst U.M., Elsener B. (2020). Laboratory tests simulating corrosion in geothermal power plants: Infuence of service conditions. Geotherm. Energy.

[55] Sherif E.S.M., Alam M.A., Al-Zahrani S.M. (2015). Fabrication of different protective coatings and studying their mechanical properties and corrosion behavior in sodium chloride solutions. Int. J. Electrochem. Sci..

[56] Yang X., Zou Y., Huang Q., Su Z., Chang X., Zhang M., Xiao Y. (2010). Improved oxidation resistance of chemical vapor reaction SiC coating modified with silica for carbon/carbon composites. J. Cent. South Univ. Technol..

[57] Li H., He Y., Luo P., Xue S., Li Z., Cheng X., Zhong J., Yan L., Fan Y. (2022). Synthesis of ZrB_2_ strengthened Ni–W composite coating and study of its mechanical characters and anti-corrosion performance. Surf. Coat. Technol..

[58] Veselý D., Kalendová A., Němec P. (2010). Properties of organic coatings depending on chemical composition and structure of pigment particles. Surf. Coat. Technol..

[59] Michel M., Ferrier E. (2020). Effect of curing temperature conditions on glass transition temperature values of epoxy polymer used for wet lay-up applications. Constr. Build. Mater..

[60] Acik G., Karabulut H.R.F., Altinkok C., Karatavuk A.O. (2019). Synthesis and characterization of biodegradable polyurethanes made from cholic acid and L-lysine diisocyanate ethyl ester. Polym. Degrad. Stab..

[61] Sabu M., Bementa E., Jaya Vins Ruban Y., Ginil Mon S. (2022). A novel analysis of the dielectric properties of hybrid epoxy composites. Adv. Compos. Hybrid Mater..

[62] Colombani J., Chauvet E., Amat S., Dupuy N., Gigmes D. (2017). A FTIR/chemometrics approach to characterize the gamma radiation effects on iodine/epoxy-paint interactions in Nuclear Power Plants. Anal. Chim. Acta.

[63] Ramírez-Herrera C.A., Cruz-Cruz I., Jiménez-Cedeño I.H., Martínez-Romero O., Elías-Zúñiga A. (2021). Influence of the Epoxy Resin Process Parameters on the Mechanical Properties of Produced Bidirectional [±45°] Carbon/Epoxy Woven Composites. Polymers.

[64] González González M., Cabanelas J.C., Baselga J., Theophanides T. (2012). Applications of FTIR on Epoxy Resins—Identification, Monitoring the Curing Process, Phase Separation and Water Uptake. Infrared Spectroscopy.

